# Time Course and Association of Functional and Biochemical Markers in Severe Semitendinosus Damage Following Intensive Eccentric Leg Curls: Differences between and within Subjects

**DOI:** 10.3389/fphys.2018.00054

**Published:** 2018-02-05

**Authors:** Gerard Carmona, Jurdan Mendiguchía, Xavier Alomar, Josep M. Padullés, David Serrano, Lexa Nescolarde, Gil Rodas, Roser Cussó, Ramón Balius, Joan A. Cadefau

**Affiliations:** ^1^Escola Superior de Ciències de la Salut, Pompeu Fabra University, Mataró, Spain; ^2^Institut Nacional d'Educació Física de Catalunya (INEFC), Universitat de Barcelona, Barcelona, Spain; ^3^Department of Physical Therapy, Zentrum Rehab and Performance Center, Barañain, Spain; ^4^Department of Radiology, Clínica Creu Blanca, Barcelona, Spain; ^5^Department of Electronic, Universitat Politècnica de Catalunya, Barcelona, Spain; ^6^Futbol Club Barcelona, Barcelona, Spain; ^7^Departament de Biomedicina, Universitat de Barcelona, Barcelona, Spain; ^8^Consell Català de l'Esport, Barcelona, Spain

**Keywords:** hamstring muscles, severe exercise-induced muscle damage, high responders, force-generating capacity, magnetic resonance imaging, creatine kinase

## Abstract

**Purpose:** To investigate the extent and evolution of hamstring muscle damage caused by an intensive bout of eccentric leg curls (ELCs) by (1) assessing the time course and association of different indirect markers of muscle damage such as changes in the force-generating capacity (FGC), functional magnetic resonance (fMRI), and serum muscle enzyme levels and (2) analyzing differences in the degree of hamstring muscle damage between and within subjects (limb-to-limb comparison).

**Methods:** Thirteen male participants performed six sets of 10 repetitions of an ELC with each leg. Before and at regular intervals over 7 days after the exercise, FGC was measured with maximal isometric voluntary contraction (MVC). Serum enzyme levels, fMRI transverse relaxation time (T2) and perceived muscle soreness were also assessed and compared against the FGC.

**Results:** Two groups of subjects were identified according to the extent of hamstring muscle damage based on decreased FGC and increased serum enzyme levels: high responders (*n* = 10, severe muscle damage) and moderate responders (*n* = 3, moderate muscle damage). In the high responders, fMRI T2 analysis revealed that the semitendinosus (ST) muscle suffered severe damage in the three regions measured (proximal, middle, and distal). The biceps femoris short head (BFsh) muscle was also damaged and there were significant differences in the FGC within subjects in the high responders.

**Conclusion:** FGC and serum enzyme levels measured in 10 of the subjects from the sample were consistent with severe muscle damage. However, the results showed a wide range of peak MVC reductions, reflecting different degrees of damage between subjects (high and moderate responders). fMRI analysis confirmed that the ST was the hamstring muscle most damaged by ELCs, with uniform T2 changes across all the measured sections of this muscle. During intensive ELCs, the ST muscle could suffer an anomalous recruitment pattern due to fatigue and damage, placing an excessive load on the BFsh and causing it to perform a synergistic compensation that leads to structural damage. Finally, T2 and MVC values did not correlate for the leg with the smaller FGC decrease in the hamstring muscles, suggesting that long-lasting increases in T2 signals after FGC markers have returned to baseline values might indicate an adaptive process rather than damage.

## Introduction

Hamstring muscles comprise the semimembranosus (SM), the semitendinosus (ST), and the biceps femoris (BF), which has a long head (BFlh) and a short head (BFsh). These muscles, particularly susceptible to injury, are required to achieve intense bursts of speed and are primarily involved in knee flexion and/or hip extension during locomotion (Yu et al., 2008; Schache et al., 2012, 2013; Morin et al., 2015).

Eccentric training has been commonly and successfully used to prevent injury (Askling et al., 2003; Goode et al., 2015; van der Horst et al., 2015). However, it is well established that high intensity (i.e., high force and/or strain) repeated eccentric contractions (i.e., lengthening muscle actions) can lead to muscle damage that requires days or weeks to recover (Clarkson and Hubal, 2002; Byrne et al., 2004; Proske and Allen, 2005; Hyldahl and Hubal, 2014). This type of damage is known as “exercise-induced muscle damage” (EIMD) (Paulsen et al., 2012). Muscle damage induced by extreme regimens of eccentric exercise presents signs such as myofibrillar disruptions (Fridén et al., 1983; Gibala et al., 2000) and myofiber necrosis (Hikida et al., 1983; Lauritzen et al., 2009). These signs are commonly assessed by histological examination of muscle tissue via biopsy; however, this technique is rarely used when hamstring muscles are involved. In such cases, EIMD symptoms are evaluated by assessing the prolonged loss of the force-generating capacity (FGC) (Byrne et al., 2004; Raastad et al., 2010). FGC seems to reflect myofibrillar disruptions, inflammation, and necrosis better than any other indirect marker of muscle damage (Paulsen et al., 2012). In this regard, a high correlation (*r* = 0.89) has been reported between the magnitude of the decrease in the maximal isometric voluntary contraction (MVC) and the proportion of muscle fibers with ultrastructural disruptions (Raastad et al., 2010). Based on the association found in several studies between decreased FGC and myofibrillar disruptions, Paulsen et al. (2012) suggested using the term “severe” EIMD when a large decrease in FGC (≥50% reduction) and/or long-lasting recovery (>1 week) were observed following muscle-damaging protocols. Although changes in the FGC provide reliable and valid information about the extent of muscle damage, it does not offer any evidence of the site of the damage, either in the muscle or the fiber structure. Therefore, when severe EIMD is likely in hamstring muscles due to intensive eccentric exercises, proxy markers of muscle damage, such as changes detected in functional magnetic resonance imaging (fMRI) and serum biochemical markers, are needed to obtain a more accurate picture of the damage.

fMRI identifies changes in metabolic activity in each muscle and provides a quantitative index of muscle activation (Cagnie et al., 2011) and damage (Larsen et al., 2007; Fouré et al., 2015). As the degree of response to intensive eccentric exercise is likely to vary in different muscles (Marqueste et al., 2008), fMRI T2 values enable the discrimination of the changes exhibited in specific muscle regions, thus offering spatially localized information (Larsen et al., 2007; Fulford et al., 2015). The higher the osmotic and pH changes within the loaded muscles during an exercise, the larger the increase in the T2 signal intensity (Schuermans et al., 2016). Studies employing fMRI have shown that the ST is the most activated hamstring muscle in the vast majority of hamstring-strengthening exercises (Mendiguchia et al., 2013a; Mendez-Villanueva et al., 2016), especially in knee-dominant exercises such as eccentric leg curls (ELCs) (Kubota et al., 2007; Mendiguchia et al., 2013b). Previous studies have shown that intensive unilateral ELCs (supramaximally loaded at 120% 1-RM) induced large changes in fMRI measurements (Kubota et al., 2007; Mendiguchia et al., 2013b) and, most importantly, large long-lasting decreases in the FGC (Kubota et al., 2007). As the ST muscle is activated the most during an ELC, it seems reasonable to assume that most of the eccentric load will fall on the ST muscle. Although the tendinous inscription could play a role in protecting the ST distal region from structural damage (van der Made et al., 2015), the extent of the damage could be exacerbated and become severe due to the excessive load.

To determine muscle damage at the cellular level, changes in the levels of serum muscle enzymes can indicate the status of fiber structures. Variations in serum muscle enzyme activities following eccentric exercises have been widely used as indirect biochemical markers of muscle damage (Brancaccio et al., 2010). Due to its mainly sarcoplasmic location in the fiber, leakage of muscle enzymes into the bloodstream has been associated with increased membrane permeability (Overgaard et al., 2002; Hyldahl and Hubal, 2014). However, large increases of muscle enzymes in the serum (i.e., >10,000 IU·L^−1^ of creatine kinase [CK]) may indicate significant damage linked to the necrosis of the whole structures of a myofibrillar segment (Lauritzen et al., 2009). Therefore, large serum increases of muscle enzymes reflect, to some extent, the amount of myofibrillar damage, especially when the damage is severe with significant ultrastructural changes (Lauritzen et al., 2009; Raastad et al., 2010). Moreover, investigating a mitochondrial-specific enzyme, such as sarcomeric mitochondrial creatine kinase (sMtCK), may offer information about the status of the mitochondria, which are especially sensitive to muscle damage and play a central role in cellular regulatory systems such as Ca^2+^ modulation and apoptosis (Newmeyer and Ferguson-Miller, 2003). Assessments of serum enzyme levels and fMRI T2 can be used together to identify the muscles from which enzymes are being released into the circulation (Larsen et al., 2007).

To better understand the effect of eccentric exercises, commonly used to prevent injury in hamstring muscles, it is important to identify the time course of the FGC, which is the most reliable indirect marker of EIMD. Moreover, it is essential to comprehend the time-related associations between the FGC and other frequently used indirect markers of EIMD, such as changes in fMRI T2 signals and serum muscle enzyme levels. Therefore, the aim of this study was to investigate the extent and evolution of hamstring muscle damage caused by an intensive bout of ELCs by (1) assessing the time course and association of different indirect markers of muscle damage such as changes in the FGC, fMRI and serum muscle enzyme levels and (2) analyzing differences in the degree of hamstring muscle damage between and within subjects (limb-to-limb comparison).

## Materials and methods

### Subjects

Thirteen healthy male students [age = 22.9 ± 2 years, height = 1.77 ± 0.6 m and weight = 74 ± 6 kg (mean ± SD)] with no history of hamstring injuries gave written informed consent to participate in the study. Because estrogen may exert an skeletal muscle protective effect (Kendall and Eston, 2002; Enns and Tiidus, 2010), maybe by increasing membrane stability and lowering CK in women (Hicks et al., 2016), female subjects were not recruited. The participant's dominant leg was determined by asking them their preferred leg when kicking a ball (Hody et al., 2013). However, there is lack of consensus in the literature on how to define lateral dominance since it can be determined according to a variety of criteria, such as strength, functional use, or performance in specific skills (Hoffman et al., 1998). The fitness level of the subjects varied according to their daily physical activity; two subjects were sedentary and the other 11 were “moderately active” to “physically active” (exercising 2–5 days·week^−1^; Table 1). The subjects had not performed systematic heavy-resistance strength training for at least 6 months prior to the experiments. Moreover, they were asked not to perform any exercise during the week before or at any time during the experimental period. The study complied with the code of ethics of the World Medical Association (Declaration of Helsinki) and was approved by the Ethics Committee of the Catalan Sports Council (*Generalitat de Catalunya*) (099S/690/2013).

**Table 1:** Physical activity levels of the study participants.

**Subject**	**Activity level**	**Sport**
1	Medium	Athletics, recreational
2	Low	Indoor football, recreational
3	Medium	Football, recreational
4	Medium	Indoor football, recreational
5	None	No exercise
6	Low	Basketball, recreational
7	High	Football, active
8	None	No exercise
9	Medium	Cycling and athletics, recreational
10	Medium	Water polo, active
11	Medium	Running, recreational
12	High	Roller hockey, active
13	Medium	Football, recreational

*Activity levels, low, two bouts of exercise per week; medium, two to four bouts of exercise per week; high, more than four bouts of exercise per week*.

### Experimental design

Changes in muscle function were examined for 1 week following a bout of ELCs performed with each leg. The recovery of the muscle FGC was assessed by repeated tests of isometric MVC during a knee flexion from a prone position. The first MVC test was performed before the eccentric exercise to establish baseline values and the rest of the tests were repeated 24, 48, and 72 h and 7 days after the exercise.

**Figure 1:**
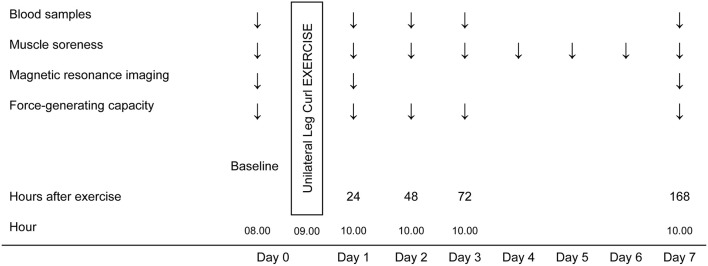
Schematic overview of the experimental design.

To monitor muscle enzyme leakage, blood samples were taken immediately before assessment of the FGC at baseline and at 24, 48, 72 h and 7 days after the exercise. To conduct a non-invasive analysis of the physiological changes occurring in the muscles involved during the exercise, fMRI was performed just before assessment of the FGC at baseline and at 24 h and 7 days after the ELCs. Finally, perceived muscle soreness in the hamstring muscles was also assessed using a visual rating scale (VRS) before and 24, 48, and 72 h and 4, 5, 6, and 7 days after the exercise protocol (Figure 1).

#### Eccentric exercise

Subjects performed six sets of 10 unilateral hamstring ELCs (Prone Leg Curl Technogym™, Italy) at 120% of their concentric 1-repetition maximum (1-RM) with each leg, with a 3-min rest between the lengthening sets. Overload was chosen according to the force-velocity properties of the skeletal muscles because they are capable of developing much higher forces in the eccentric phase of conventional resistance exercise compared when they contract concentrically (Reeves et al., 2009; Franchi et al., 2014, 2015). The 1-RM assessment is described elsewhere (Mendiguchia et al., 2013b). During the exercise protocol, subjects were instructed to lower the weight from a knee-flexed position (~100°) to a knee-extended position (0°) in 3 s, trying to maintain the lowering velocity as constant as possible with plantar flexion of the ankle to minimize gastrocnemius muscle contribution. The subjects were verbally encouraged to produce maximal force at the starting position and to resist maximally against the knee-extending action throughout the range of motion. The weight was raised after each eccentric repetition by two examiners to ensure that the exercise was an eccentric-only task for the subject.

#### Force-generating capacity

FGC was measured as the MVC, i.e., the average force in a 1-second window when a force plateau has been established (Tesch et al., 2004). The MVC of the hamstring muscles was measured for each leg with a force gauge connected to an A/D converter system, MuscleLab 4020e (Ergotest AS, Langesund, Norway). Subjects were prone with the hip joint at 40° of flexion and the knee joint at 30° of flexion, and were verbally encouraged during the test to ensure maximal effort. Subjects performed two isometric MVCs of 3–5 s, with a 1-min rest between the contractions. If any countermovement was evident, an additional MVC was measured. According to the percentage of reduction of the FGC and its time course, the muscle damage was classified as severe EIMD (i.e., large reduction in the MVC of >50% of the baseline values and/or recovery not completed in 1 week) or moderate EIMD (i.e., notable MVC decreases of 20–50% of the baseline values and recovery completed between 48 h and 7 days; Paulsen et al., 2012) during the repeated MVC tests following the eccentric exercise. The leg with the larger MVC reduction was considered to have more damaged hamstring muscles (ST and BF [BFlh and BFsh]).

#### Muscle soreness

A 10-point VRS was used to quantify muscle soreness in the hamstring muscles. Each number on the scale was accompanied by descriptive words for soreness, from 0 indicating no soreness to 10 indicating intolerably intense soreness. Subjects stretched and contracted (isolated unloaded knee flexion from a prone position) to assess general soreness in the hamstring muscles.

#### Blood analysis

An 8-mL blood sample was drawn from an antecubital vein. The blood was allowed to clot for 30 min at room temperature (21°C) and was then centrifuged at 3,000 g for 10 min at 4°C. After separation, serum aliquots were stored at −80°C until analysis. Measurements of creatine kinase (CK), myoglobin, lactate dehydrogenase (LDH), aspartate aminotransferase (AST), alanine aminotransferase (ALT), gamma-glutamyltransferase (γGT), and alkaline phosphatase (ALP) were performed on an Advia 2400 device (Siemens™ Medical Solutions Diagnostics, Tarrytown, NY, USA). Serum concentration of sMtCK was measured by a commercial ELISA kit SEC386Hu (Cloud Clone Corp., Houston, TX, USA), according to the manufacturer's protocol.

#### Magnetic resonance imaging

fMRI (3T scanner; Siemens, Erlangen, Germany) was performed ~30 min before and 24 h and 7 days following the exercise. Subjects were supine on the MR gurney with the head outside the MR bore and thighs covered with one 32- and two flexible 4-channel coils in the proximal and distal segments, respectively. A custom-made foot restraint was used to standardize and fix the limb position, and to avoid any compression of the thigh muscles. Twelve cross-sectional images of the thigh of both legs were obtained, starting at the very distal margin of the ischial tuberosity and using the following scan sequences: (a) axial fat-suppressed proton density, TR 3,000 ms, TE 30-33, eco train 4, slice thickness 3.5 mm, gap 28 mm, FOV 400 × 290 mm, matrix 320 × 180, and ipat 2; and (b) axial T2 mapping, TR 1,000 ms, TE 18, 36, 54, 72, 90, and 108, eco train 6, FOV 400 × 400 mm, matrix 256 × 256, slice thickness 3.5 mm, and gap 28 mm. A parametric image was generated from the T2 mapping sequence using the Leonardo workstation (Siemens, Erlangen, Germany). Scout images and anatomical landmarks were obtained to ensure identical positioning at baseline and post-exercise scans.

Since no T2 value changes have previously been reported in any regions of the SM following ELCs (Kubota et al., 2007; Mendiguchia et al., 2013b) and as the present investigation focused on the interaction between the ST and BF muscles, T2 of only the ST, BFlh, and BFsh muscles of both legs were measured using the eFilm Lite software version 3.1 (Merge Healthcare, Chicago, IL). Using the fat-suppressed images to detect any confounding artifact (i.e., vessels and fat), a circular region of interest (ROI) was selected for individual hamstring muscles in each of the T2 mapping images where muscles were visible. Following pre-exercise scan analysis, the same circular ROIs were placed in the T2 images of the post-exercise scans to ensure the same positioning. In the evaluations, images containing areas at 30% (proximal), 50% (middle), and 70% (distal) of the length of the thigh from the upper border of ischial tuberosity (0%) to the lower border of the tibial plateau (100%) were used (Kubota et al., 2007). The same researcher performed the MRI scans and T2 calculations. High inter-tester reliability, with intra-class correlation coefficients ranging from 0.87 to 0.94, has previously been reported (Cagnie et al., 2011).

### Statistics

Distributions were considered for each of the variables, with the normality of continuous variables assessed by the Shapiro-Wilk test. Asymmetrically-distributed variables were log transformed before analysis. One-way repeated-measures ANOVA was used to identify the effect of time on serum enzyme levels. If significant effects were found, a *post-hoc* Bonferroni-corrected paired *t*-test was applied to identify significant differences from baseline values. A two-way repeated-measures ANOVA (leg dominance × time) was performed to identify the main effects of leg dominance on the MVC. A two-way repeated-measures ANOVA [leg (leg that showed the larger and leg that showed the smaller decrease in FGC) × time] followed by a paired *t*-test with a Bonferroni correction was performed to identify statistically significant differences from baseline MVC and muscle soreness values as well as differences between legs following ELCs. A three-way repeated-measures ANOVA [leg muscle (leg that showed the larger and leg that showed the smaller decrease in FGC) × region × time] followed by a paired *t*-test with a Bonferroni correction was performed to identify statistically significant differences from the baseline T2 values and differences between the hamstring muscles and their respective measured regions (proximal, middle, and distal) after the exercise protocol. Differences between the groups of subjects (high and moderate responders) were assessed using the Mann-Whitney U test. Associations between variables of interest were assessed using Spearman's rank correlation coefficient. Data are presented as mean ± standard error of the mean (SEM), unless otherwise stated. The level of significance was set at *P* < 0.05. Statistical analysis was performed with SPSS version 20.0 (SPSS Statistics, IBM Corp., Armonk, NY, USA).

## Results

### Force-generating capacity

**Figure 2:**
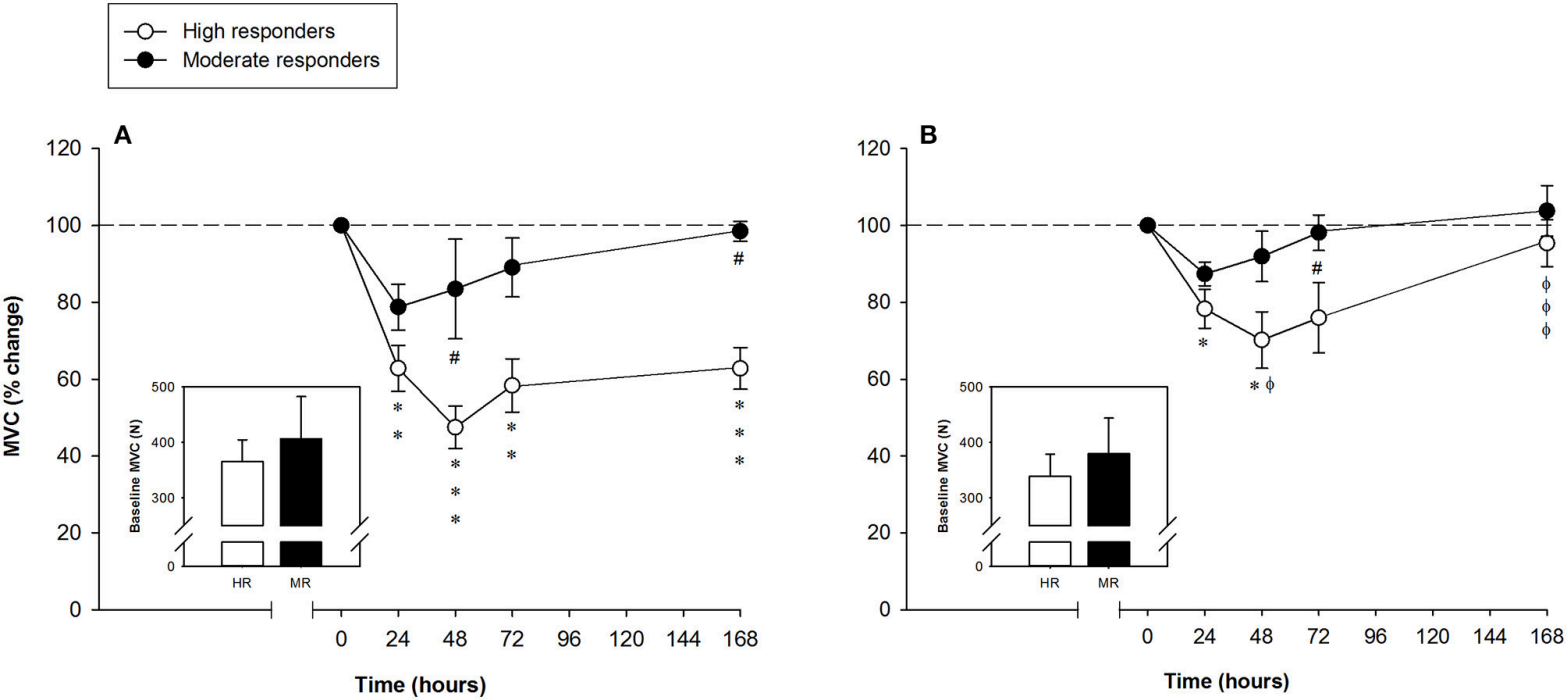
Mean (± SEM) percentages of the maximal voluntary contraction (MVC) of the hamstring muscles of the leg that showed the **(A)** larger and **(B)** smaller reduction of the force-generating capacity following the exercise protocol. Mean (± SEM) values of the baseline MVC are shown in the inset graphs. ^*^, ^**^, and ^***^Indicate a significant difference from the baseline value at *P* < 0.05, *P* < 0.01, and *P* < 0.001, respectively. ^#^Indicates a significant difference between the groups at *P* < 0.05. ^ϕ^ and ^ϕϕϕ^Indicate a significant difference between legs of the high responders at *P* < 0.05 and *P* < 0.001, respectively.

According to the MVC data, muscle damage was classified as severe or moderate following the criteria presented above (see Materials and Methods). Subjects were then grouped as “high” (*n* = 10) or “moderate” (*n* = 3) responders according to the degree of hamstring muscle [ST and BF (BFlh and BFsh)] damage. Except in subjects 5, 6, and 8, the leg with the larger decrease in the FGC of the hamstring muscles was the non-dominant one, but there was no significant effect of leg dominance (dominant or non-dominant) on measured variables. No baseline differences in the MVC were found between (high and moderate responders) or within (leg-to-leg comparison in the high responders) subjects (Figure 2, inset graphs). The time-related changes in the MVC of the high and moderate responders are shown in Figure 2. Legs showing the greater FGC reduction of the hamstring muscles in the high responders suffered significant MVC decreases of 34 ± 7%, 52 ± 7%, 39 ± 8%, and 38 ± 7% at 24, 48, 72 h and 7 days after the ELCs, respectively. Unexpectedly, among the high responders, the leg with the smaller reduction in the MVC of the hamstring muscles only presented statistically significant decreases of 18 ± 6% and 34 ± 9% at 24 and 48 h, respectively, the percentage of MVC reduction differing significantly between legs at 48 h and 7 days after the ELCs (Figure 2). The three moderate responders (subjects 1, 7, and 9) showed a surprisingly low FGC reduction in both legs, with significant differences in the MVC reductions between the high and moderate responders occurring 48 h and 7 days after the exercise regimen (Figure 2).

### Muscle soreness

**Figure 3:**
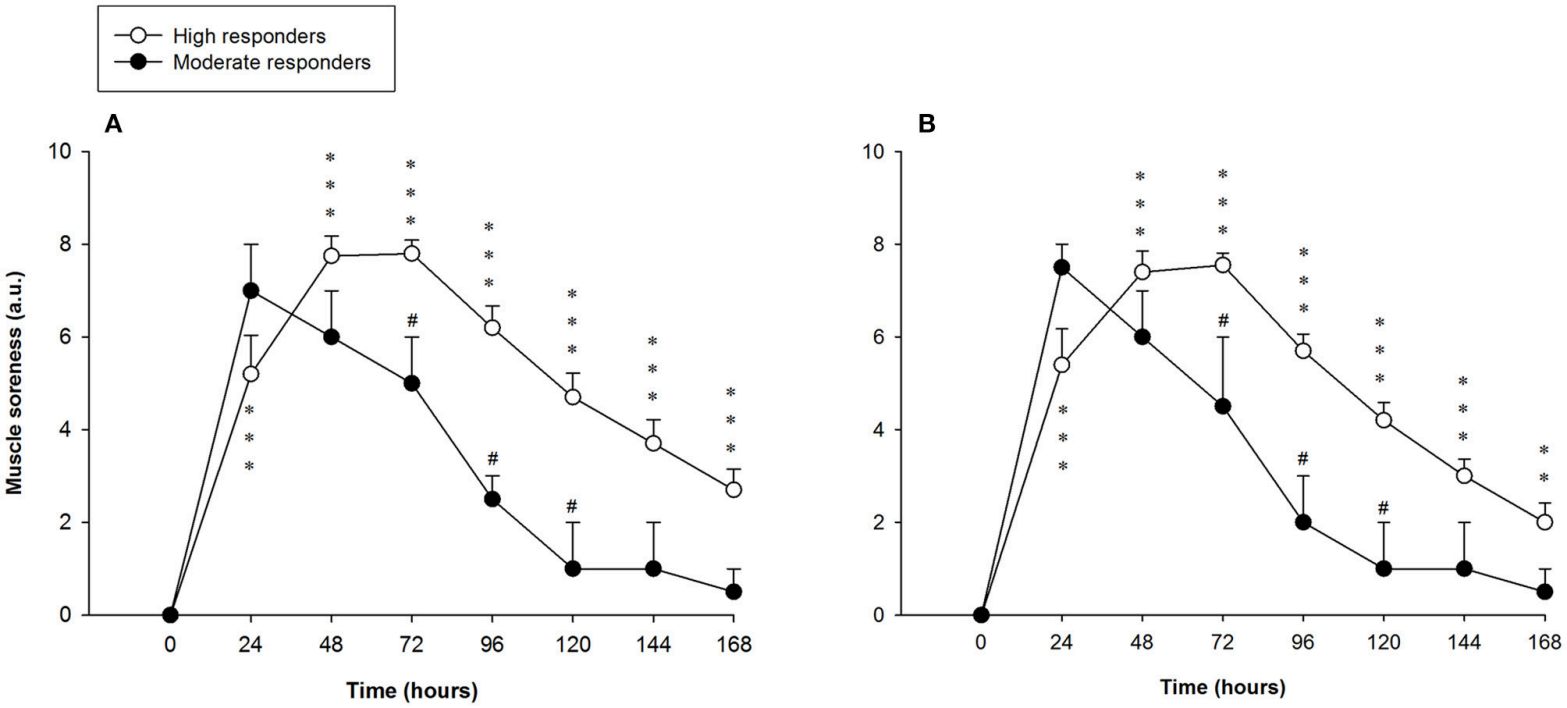
Mean (± SEM) values (arbitrary units, a.u.) of perceived muscle soreness of the hamstring muscles of the leg that showed the **(A)** larger and **(B)** smaller reduction of the force-generating capacity following the exercise protocol. ^*^, ^**^, and ^***^Indicate a significant difference from the baseline value at *P* < 0.05, *P* < 0.01, and *P* < 0.001, respectively. ^#^Indicates a significant difference between the groups at *P* < 0.05.

Perceived muscle soreness among the high responders during stretching and contracting (isolated unloaded knee flexion from a standing position) was significantly elevated over baseline values at every time-point analyzed. Muscle soreness increased after the exercise protocol and peaked at 72 h, with a value of 7.8 ± 0.3 arbitrary units (a.u.). Significant differences in muscle soreness were found between the high and moderate responders at 72, 96 and 120 h after the exercise (Figure 3). There were no differences within subjects (leg-to-leg comparison in the high responders). Correlations between muscle soreness and peak and long-lasting MVC reductions observed 48 h and 7 days after exercising are shown in Table 2.

**Table 2:** Spearman's rank correlation coefficient matrix of force-generating capacity (isometric maximum voluntary contraction), serum enzyme levels, and muscle soreness (*n* = 13).

		**Force-generating capacity**			
		**LL FGC**		**SL FGC**	
		**MVC 48 h**	**MVC 7 d**	**MVC 48 h**	**MVC 7 d**
Serum	CK 48 h	−0.720^**^	–	−0.682^*^	–
enzymes	CK 7 d	–	−0.566^*^	–	−0.126
	sMtCK 48 h	−0.691^**^	–	−0.570	–
	sMtCK 7 d	–	−0.572^*^	–	−0.278
	Myoglobin 48 h	−0.703^**^	–	−0.673^*^	–
	Myoglobin 7 d	–	−0.322	–	−0.262
	LDH 48 h	−0.654^*^	–	−0.664^*^	–
	LDH 7 d	–	−0.347	–	−0.083
	AST 48 h	−0.753^**^	–	−0.582	–
	AST 7 d	–	−0.549	–	−0.214
	ALT 48 h	−0.698^**^	–	−0.664^*^	–
	ALT 7 d	–	−0.578^*^	–	−0.231
Muscle soreness	LL FGC VRS 48 h	−0.830^**^	–	–	–
	LL FGC VRS 7 d	–	−0.061	–	–
	SL FGC VRS 48 h	–	–	−0.788^**^	−
	SL FGC VRS 7 d	–	–	–	0.186

*Note that the data are presented for the legs showing the larger (LL FGC) and smaller (SL FGC) reduction of force-generating capacity following the exercise protocol. CK, Creatine kinase; sMtCK, sarcomeric mitochondrial creatine kinase; LDH, lactate dehydrogenase; AST, aspartate aminotransferase; ALT, alanine aminotransferase; VRS, visual rating scale*.

* and ** *indicate a significant correlation at P < 0.05 and P < 0.01, respectively*.

### Serum muscle enzymes

#### Creatine kinase

**Figure 4:**
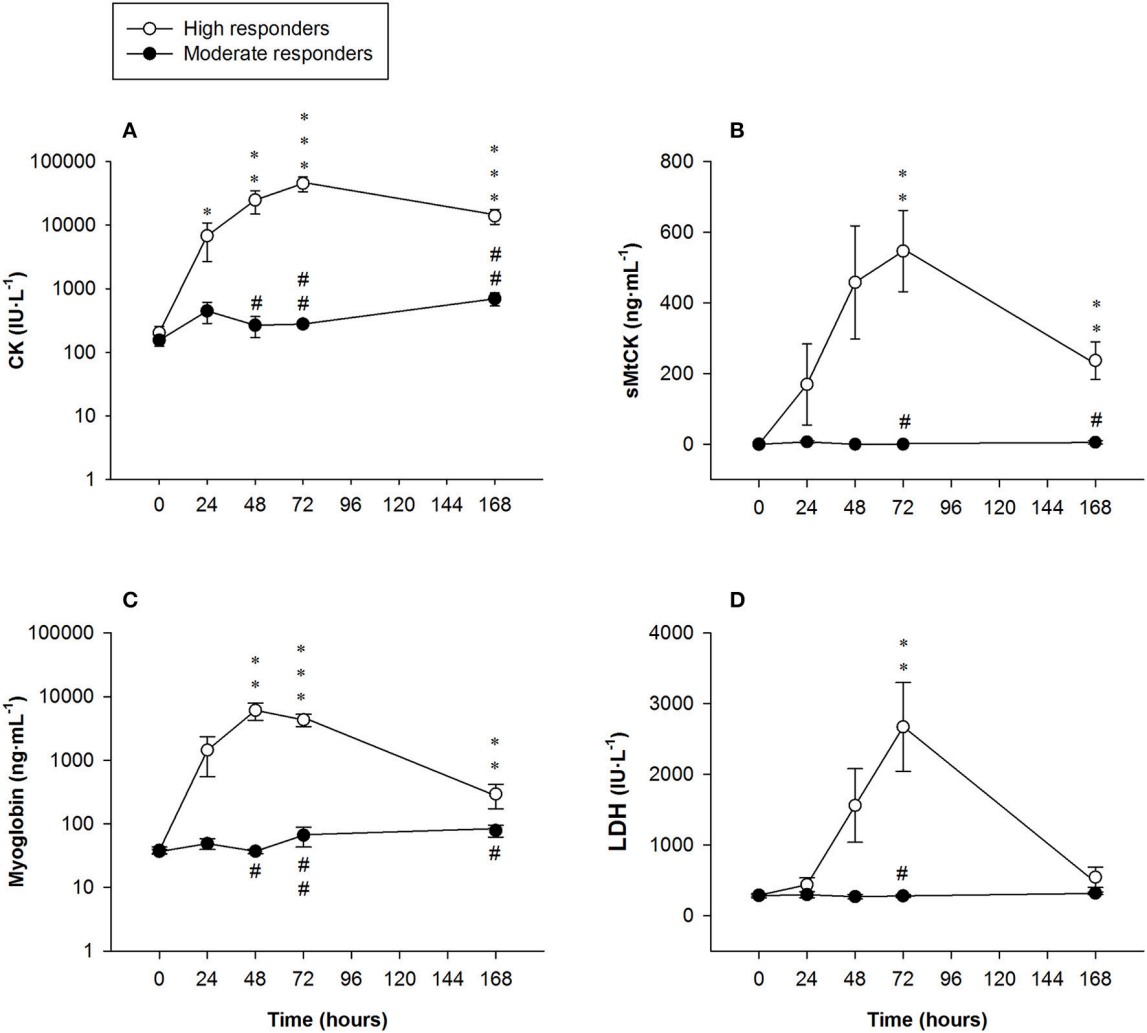
Mean (± SEM) values of **(A)** creatine kinase (CK, note the logarithmic scale on the y-axis); **(B)** sarcomeric mitochondrial creatine kinase (sMtCK); **(C)** myoglobin (note the logarithmic scale on the y-axis) and **(D)** lactate dehydrogenase (LDH). ^*^, ^**^, and ^***^Indicate a significant difference from the baseline value at *P* < 0.05, *P* < 0.01, and *P* < 0.001, respectively. ^#^ and ^##^Indicate a significant difference between the groups at *P* < 0.05 and *P* < 0.01, respectively.

Serum CK levels of the high responders sharply increased until it peaked 72 h after exercising [45,455 ± 9,922 U·L^−1^ (range: 530–95,920 U·L^−1^)], decreasing 7 days after the ELCs [13,990 ± 3,622 U·L^−1^ (range: 4,198–42,972 U·L^−1^)]. Serum CK levels of the moderate responders followed a biphasic pattern, increasing 24 h after exercising, declining slightly at 48 h before increasing again until it peaked 7 days after exercising [701 ± 163 U·L^−1^ (range: 447–1,005 U·L^−1^)]. There were significant differences in the serum CK levels between the two groups at 48, 72 h, and 7 days after the ELCs (Figure 4).

#### Sarcomeric mitochondrial creatine kinase

Serum sMtCK concentrations in the high responders increased until it peaked at 72 h [547 ± 115 ng·mL^−1^ (range: 0–918 ng·mL^−1^)] and remained significantly elevated 7 days after exercising [237 ± 53 ng·mL^−1^ (range: 76–616 ng·mL^−1^)]. Significant differences in serum sMtCK levels were found between the high and moderate responders at 72 h and 7 days after exercising (Figure 4).

#### Myoglobin

Myoglobin concentrations in the high responders largely exceeded clinically normal values (<100 ng·mL^−1^), peaking at 48 h [6,085 ± 1,850 ng·mL^−1^ (range: 55–13,618 ng·mL ^−1^)] before markedly decreasing 7 days following the exercise regimen [293 ± 121 ng·mL^−1^ (range: 88–1,353 ng·mL^−1^)]. Significant differences were found between the groups from 48 h onwards (Figure 4).

#### Lactate dehydrogenase

Serum LDH activity in the high responders was higher than the clinically normal range (250–450 IU·L^−1^), peaking at 72 h [2,670 ± 628 IU·L^−1^ (range: 301–5,145 IU·L ^−1^)] and then decreasing notably 7 days after exercising. Significant differences between the groups were only observed at 72 h (Figure 4).

#### Aspartate aminotransferase

**Figure 5:**
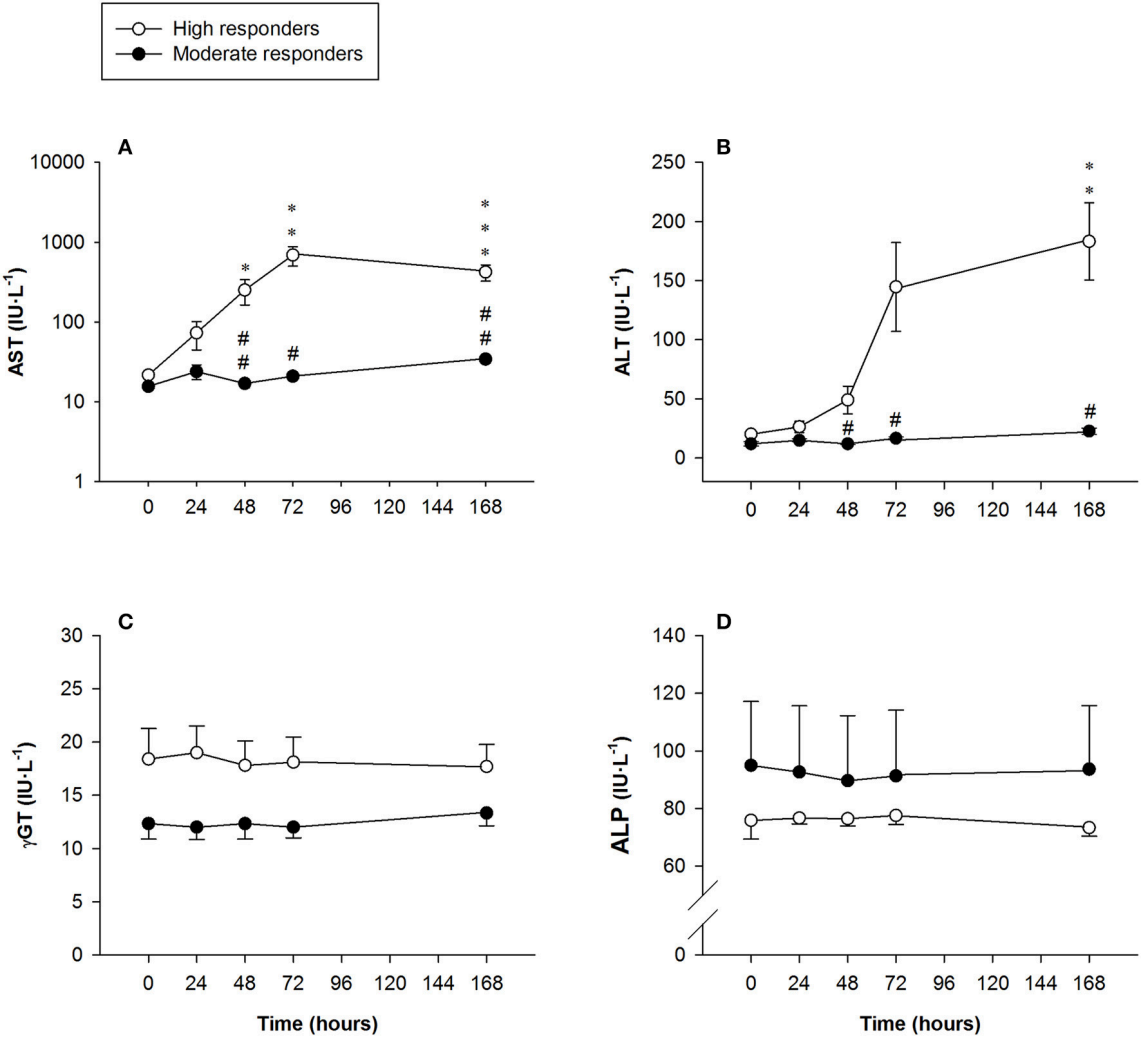
Mean (± SEM) values of **(A)** aspartate aminotransferase (AST, note the logarithmic scale on the y-axis); **(B)** alanine aminotransferase (ALT); **(C)** gamma-glutamyltransferase (γGT), and **(D)** alkaline phosphatase (ALP). ^*^, ^**^, and ^***^Indicate a significant difference from the baseline value at *P* < 0.05, *P* < 0.01, and *P* < 0.001, respectively. ^#^ and ^##^Indicate a significant difference between the groups at *P* < 0.05 and *P* < 0.01, respectively.

AST activity in the high responders was above the clinically normal range (5–40 IU·L^−1^), peaking at 72 h [691 ± 191 IU·L^−1^ (range: 21–1,813 IU·L^−1^)] and remaining significantly higher than baseline values 7 days after the exercise protocol [422 ± 96 IU·L^−1^ (range: 132–1,097 IU·L^−1^)]. Significant differences were found between the high and moderate responders at 48 h, 72 h and 7 days after exercising (Figure 5).

#### Alanine aminotransferase

Serum ALT activity in the high responders showed a sharp increase above the clinically normal range (5–40 IU·L^−1^) at 72 h, declining somewhat but still being statistically significantly higher 7 days after exercising [183 ± 191 IU·L^−1^ (range: 23–365 IU·L^−1^)]. Significant differences in ALT activity were observed between the groups at 48 h, 72 h and 7 days after exercising (Figure 5).

#### Gamma-glutamyltransferase

There were no changes in serum γGT activity in any of the subjects at the time-points measured (Figure 5).

#### Alkaline phosphatase

No changes were observed in serum ALP activity in any of the subjects at the time-points measured (Figure 5).

Correlations between serum enzyme levels and peak and long-lasting MVC reductions observed 48 h and 7 days after the ELC protocol are shown in Table 2.

We performed laboratory tests to discard acute renal failure in three of the high responders who reported changes in their urine from a clear to a brownish color. It is, however, important to state that the recovery observed 3 days following the exercise regimen was a general trend for all the high responders.

### Functional magnetic resonance imaging

Significant T2 increases of 146, 172, and 186% were found in all the sections of the ST muscle [proximal (30%), middle (50%), and distal (70%), respectively] among the high responders 7 days after the exercise Table 3. Moderate T2 increases of 67% revealed that the BFsh muscle in the high responders was also damaged 7 days after the ELC regimen. There were no T2 differences in the BFlh muscle with regard to baseline values. Significant differences between the high and moderate responders were found in the ST T2 values from both legs 7 days after exercising. Surprisingly, there were no T2 differences between the legs showing the larger and lower FGC reduction of the hamstring muscles following the ELC bout Table 3. Analyses of variance revealed no T2 differences among the ST muscle regions assessed. Finally, the T2 values obtained 7 days after the exercise protocol from all the ST sections of the leg with the larger FGC decrease of the hamstring muscles correlated with the reductions in the MVC, but there was no such correlation in the leg with the smaller FGC decrease in the hamstring muscles (Figure 6). Furthermore, there were no correlations between the MVC reductions and the BFsh T2 values.

**Table 3:** Mean values (± SEM) of fMRI T2 signals from the hamstring muscles before and after unilateral eccentric leg curls.

			**T2 (ms)**	
			**Leg**	
**Muscle (section)**	**Time**	**Group**	**LL FGC**	**SL FGC**
BFsh	Baseline	High responders (*n* = 10)	43.4 ± 2.2	45.7 ± 1.5
(70%, distal)	24 h		49.8 ± 2.6	60.2 ± 4.2^***^
	7 d		72.6 ± 9.6^*^	73.7 ± 11.6^*^
	Baseline	Moderate responders (*n* = 3)	47.2 ± 9.3	47.9 ± 7.7
	24 h		50.8 ± 9.7	49.9 ± 8.1
	7 d		48.0 ± 8.5	46.7 ± 7.4
ST	Baseline	High responders (*n* = 10)	48.0 ± 1.7	51.8 ± 1.9
(30%, proximal)	24 h		110.7 ± 42.3	117.3 ± 25.6
	7 d		117.9 ± 14.6^***^	154.0 ± 19.8^***^
	Baseline	Moderate responders (*n* = 3)	44.8 ± 9.1	52.6 ± 8.1
	24 h		54.8 ± 12.8	68.4 ± 10.6
	7 d		49.6 ± 18.9^#^	59.7 ± 9.1^#^
ST	Baseline	High responders (*n* = 10)	44.4 ± 1.9	43.2 ± 2.3
(50%, middle)	24 h		113.4 ± 48.3	89.4 ± 17.9
	7 d		119.8 ± 20.0^***^	122.9 ± 15.0^***^
	Baseline	Moderate responders (*n* = 3)	43.2 ± 8.2	46.9 ± 7.5
	24 h		49.5 ± 9.3	53.5 ± 8.5
	7 d		43.7 ± 14.9^##^	45.3 ± 6.8^#^
ST	Baseline	High responders (*n* = 10)	43.5 ± 0.8	46.3 ± 1.9
(70%, distal)	24 h		62.4 ± 8.7	72.8 ± 8.7
	7 d		124.6 ± 21.2^***^	143.2 ± 17.7^***^
	Baseline	Moderate responders (*n* = 3)	43.7 ± 8.6	48.2 ± 7.5
	24 h		47.1 ± 9.1	49.1 ± 7.5
	7 d		44.0 ± 17.9^##^	45.3 ± 6.0^#^

*Only the T2 values from the hamstring muscle sections showing significant time-related changes from baseline values are shown (biceps femoris short head, BFsh, and semitendinosus, ST). T2 values of the hamstring muscles of the leg showing the larger (LL FGC) and smaller (SL FGC) reduction of force-generating capacity following the exercise protocol are shown*.

*, **, and *** *Indicate a significant difference from the baseline value at P < 0.05, P < 0.01, and P < 0.001, respectively*.

# and ## *Indicate a significant difference between the groups at P < 0.05 and P < 0.01, respectively. Note that there were no differences between the ST regions and no differences between legs*.

**Figure 6:**
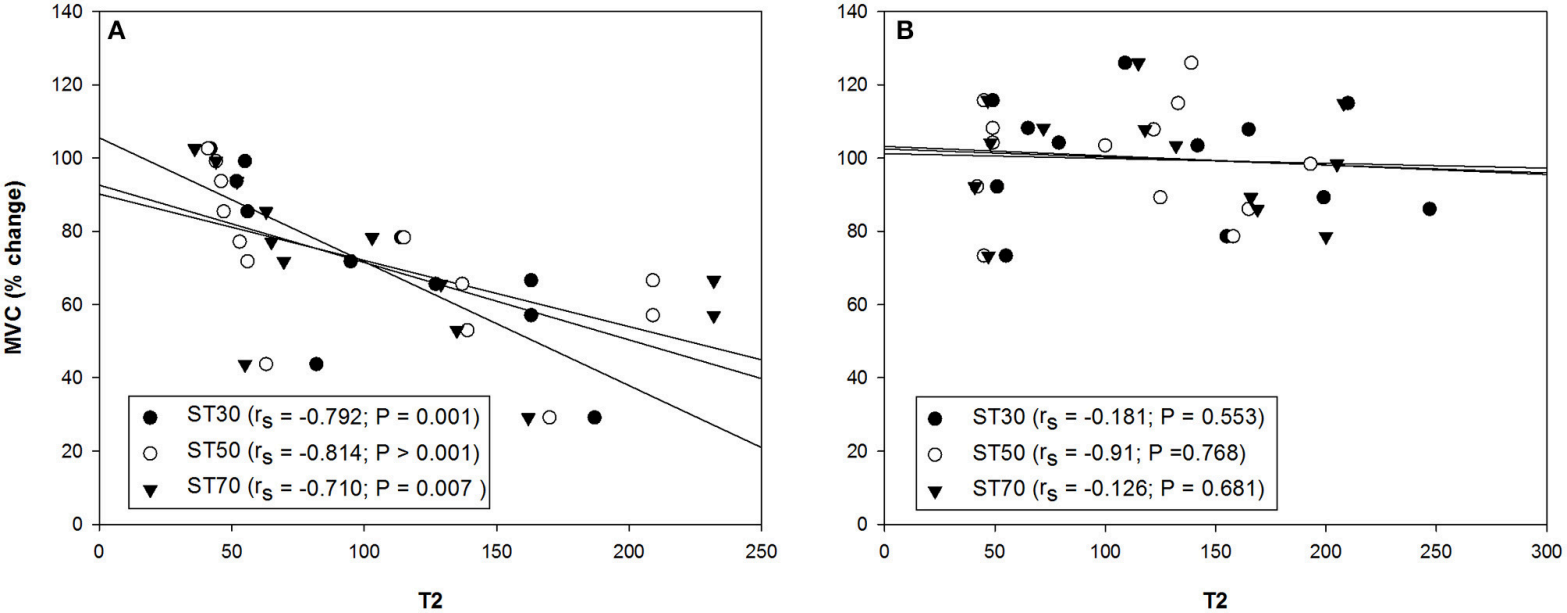
Correlation between the percentage of change in the maximal voluntary contraction (MVC) and semitendinosus (ST) muscle section (30%, proximal; 50%, middle and 70%, distal) T2 values from the hamstring muscles of the leg with the **(A)** larger and **(B)** smaller reduction of the force-generating capacity 7 days after the exercise protocol (*n* = 13). *r*_*s*_, Spearman's rank correlation coefficient.

## Discussion

The aim of this study was to investigate the extent and evolution of hamstring muscle damage caused by an intensive bout of ELCs. While the results of the changes in the FGC and serum enzyme levels were used as indirect markers to determine the extent of muscle and cellular damage (Warren et al., 1999; Hubal et al., 2007; Brancaccio et al., 2010; Paulsen et al., 2012), fMRI T2 values provided spatially localized information by indicating the changes occurring in specific muscle regions (Larsen et al., 2007; Fulford et al., 2015).

### Force-generating capacity

**Figure 7:**
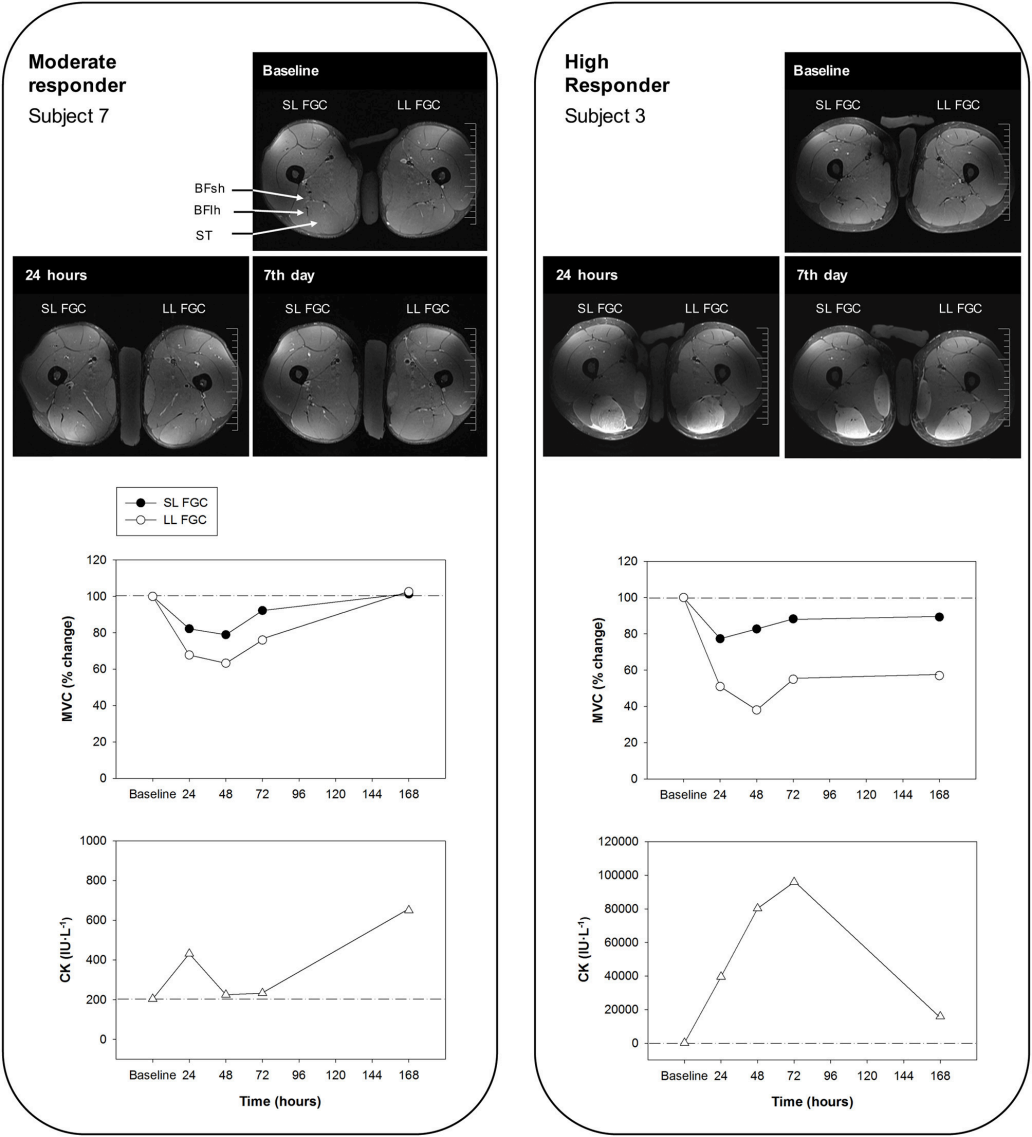
Representative T2-weighted magnetic resonance images of the middle ST section from a moderate responder (subject 7) **(left)** and a high responder (subject 3) **(right)** before and 24 h and 7 days after the eccentric exercise (BFlh, biceps femoris long head; BFsh, biceps femoris short head; and ST, semitendinosus). Percentages of maximal voluntary contraction (MVC) of the hamstring muscles of the leg showing the larger and smaller reductions of force-generating capacity (LL FGC and SL FGC, respectively) and values of serum creatine kinase (CK) levels at baseline and at regular intervals over 7 days after exercising are shown. Note that both subjects play the same sport (football), but present different levels of activity (high versus medium; Table 1).

We first classified the 13 subjects into two groups based on the leg that suffered the larger decrease in MVC following intensive ELCs. This is a reliable measure that is considered the best indirect marker of the extent of EIMD (Warren et al., 1999; Hubal et al., 2007; Paulsen et al., 2012). Two groups of subjects were identified according to the extent of EIMD, which was indicated by peak reductions in the FGC (reflecting the number of fibers with ultrastructural and myofibrillar disruptions (Lieber et al., 1994; Lauritzen et al., 2009; Raastad et al., 2010; Paulsen et al., 2012) and the time required for recovery. The high responders (*n* = 10) showed an average peak MVC loss of 52% and/or recovery that was not completed in 1 week (severe EIMD), while the moderate responders (*n* = 3) presented an average peak MVC loss of 21% and/or recovery that was achieved in 2–7 days (moderate EIMD) (Figure 7). Large reductions in the hamstring FGC among the high responders suggest that severe muscle damage can be induced in humans following voluntary contractions (Lauritzen et al., 2009).

### Functional magnetic resonance imaging

#### Severe uniform semitendinosus damage

Hamstring exercises relying on knee flexion induced more noticeable ST T2 increases and, hence, ST muscle use (Mendiguchia et al., 2013a,b; Fernandez-Gonzalo et al., 2016). As expected, the fMRI T2 results from the present investigation suggested that the individual responses of the hamstring muscles differed following ELCs. For example, the ST muscle showed the highest and most long-lasting T2 increases following the exercise protocol. Uniform T2 changes were found across all the ST regions measured [proximal, middle, and distal (30–50–70% of muscle length, respectively)] 7 days after intensive ELCs in the high responders. Along with the long-lasting FGC decreases 7 days after exercising, these results indicate that all three ST muscle regions measured suffered a similar severe degree of damage. Although a previous study (Kubota et al., 2007) has reported non-uniform changes among the same three ST regions following ELCs, these differences were only found 48 and 72 h after the exercise. As in our investigation, the previous study found uniform changes across the three ST regions 24 h and, most importantly, 7 days after ELCs. Although ST architecture and innervation display certain singularities (Woodley and Mercer, 2005; van der Made et al., 2015), when eccentric load becomes excessive, large amounts of the load are placed on the proximal and distal regions, inducing excessive tensile and shear stress that exacerbates the damage in the vast majority of the ST muscle. Therefore, we postulate that the tendinous inscription does not play a role in protecting the ST muscle against severe damage under intensive eccentric loading during knee flexions. Moreover, the morphological properties of the ST muscle may contribute to a more efficient performance of an eccentric knee flexion (Mendiguchia et al., 2013b). Hence, it can be concluded that the ST muscle seems to be the most suited hamstring muscle for performing an ELC.

#### Biceps femoris short head synergistic compensation

Significant T2 increases (smaller compared to those from the ST muscle) were also observed in the BFsh of the high responders. These fMRI changes, together with the large long-lasting FGC reductions, suggested that this muscle was also damaged following intensive ELCs. It has been suggested that the ST and BFsh play a prominent role in producing and controlling the torques around the knee joint under high loading conditions (Schuermans et al., 2014), being activated the most during ELCs and Nordic hamstring exercises (Kubota et al., 2007; Mendiguchia et al., 2013b). The BFsh is a uniarticular hamstring muscle that crosses only the knee joint and has a long fascicle, like the ST, and a small physiological cross-sectional area compared to the other hamstring muscles (Barrett, 1962; Wickiewicz et al., 1983; Friederich and Brand, 1990; Woodley and Mercer, 2005). The BFsh and ST muscles are designed to generate forces over great lengths and can be both categorized as excursion muscles. The anatomical characteristics of the BFsh muscle enable it to perform a synergistic compensation to accomplish the eccentric knee flexion more efficiently during an ELC. When the ST cannot maintain its strength and performance (exhibiting little effort compared to what it is supposed to generate), the BFsh is loaded with an excessive tensile shear that leads to structural damage. ELCs did not induce noticeable damage on the ST and BFsh muscles in the moderate responders, probably because their hamstring muscles could maintain the efficiency of intermuscular coordination. Therefore, it appears that the ST and BFsh display a complex connection based on a synergistic activation and recruitment pattern that indicates high interdependence in terms of the magnitude of muscle loading and the adequacy of muscle functioning (Schuermans et al., 2014).

### Serum enzymes

There were significant differences in serum enzyme activities or concentrations following the ELC regimen between the high and moderate responders. The severe ST muscle damage in the high responders was further highlighted by the clear increases in serum muscle enzyme levels. Serum CK elevation, to some extent, reflects the amount of myofibrillar damage, especially when the damage is severe (Lauritzen et al., 2009; Raastad et al., 2010). Moreover, large increases in serum CK and myoglobin levels 4–5 days after exercising may indicate degradation of protein structures and segmental myofiber necrosis (Brancaccio et al., 2010; Paulsen et al., 2012). The correlation between CK serum levels and MVC (FGC decreases) 7 days after the exercise suggests that the long-lasting increases in CK levels were related to myofiber necrosis. The CK increases in the high responders 7 days after the intensive ELCs (range: 4,198–42,972 U·L^−1^) were similar to the CK elevation observed in exertional rhabdomyolysis (Kenney et al., 2012). However, exertional rhabdomyolysis was excluded since eccentric exercises can produce marked elevations in serum enzyme levels without renal compromise (Clarkson et al., 2006; Scalco et al., 2016) and we also found no laboratory evidence of acute renal failure in any of the three subjects who reported dark urine following the ELC regimen. Although hypertransaminasemia was also observed, hepatic damage was also ruled out because the hepatic injury markers ALP and γGT remained unchanged after the intensive ELCs in all the subjects (Bessa et al., 2008). AST and ALT are ubiquitously present in most tissues, including skeletal muscle (Botros and Sikaris, 2013; Dahlqvist et al., 2013), and hypertransaminasemia is commonly present in patients with high CK levels resulting from extreme exercise regimens (Nathwani et al., 2005). We can therefore deduce that the observed hypertransaminasemia following ELCs is linked to muscle damage.

A small biphasic pattern in CK, myoglobin, AST and ALT levels was observed in the moderate responders. An earlier study reported a biphasic enzymatic response, but only in high responders who suffered severe muscle damage after eccentric exercises (Paulsen et al., 2010). This observed biphasic pattern could be related to an initial increase in sarcolemmal permeability produced by mechanical straining during an exercise, with the following peak in serum enzyme levels 7 days after the ELCs due to segmental myofiber necrosis (Lauritzen et al., 2009; Paulsen et al., 2010). The serum enzyme levels in the moderate responders 7 days following intensive ELCs could be due to minor myofiber necrosis since minimal sarcomere disruptions can occur without significant changes in the FGC (Gibala et al., 2000), probably because force can be transmitted laterally through neighboring myofibrils and the extracellular matrix (Bloch and Gonzalez-Serratos, 2003; Grounds et al., 2005).

Serum increases of sMtCK were of particular interest because, in contrast to the other enzyme markers, sMtCK levels were absent or only marginally increased in the serum of the moderate responders. Large increases of serum myoglobin might be linked to mitochondrial damage because the release of iron ions from the haem of myoglobin promotes the peroxidation of mitochondrial membranes (Plotnikov et al., 2009). Mitochondria are key regulators of many cellular processes like Ca^2+^ regulation and apoptosis (Newmeyer and Ferguson-Miller, 2003). The mitochondrial intermembrane space contains several pro-apoptotic proteins that can lead to cell death upon release into the sarcoplasm (Primeau et al., 2002). sMtCK acts as an energy sensor, coupling the cellular energy state to cell apoptosis (Schlattner et al., 2006). Apoptosis has been suggested to be involved in muscle remodeling and repair following electrically-induced eccentric contractions in rats (*Wistar*) (Biral et al., 2000). The significant increases in serum sMtCK found in the high responders are likely to be indicative of mitochondrial swelling and reduced muscle respiratory capacity (Chen et al., 2000). Given the correlations observed between serum sMtCK levels and MVC reductions 48 h and especially 7 days after intensive ELCs, FGC reductions could indicate the number of myofibrils that have died by either apoptosis (due to a process triggered by large increases in sMtCK) and/or necrosis. Moreover, prolonged exercise has been demonstrated to induce mitochondrial damage in rats (*Sprague-Dawley*), with the training status having a protective effect on the mitochondria against exercise (Chen et al., 2000) via a specific reduction of mitochondrial Ca^2+^ uptake (Bonner et al., 1976) and increased antioxidant capacity (Venditti and Di Meo, 1997). Although only observed in animal models (*Sprague-Dawley* rats) (Chen et al., 2000), it seems reasonable to assume that the training status of the moderate responders might confer a protective effect on the mitochondria against eccentric EIMD.

### Moderate responders: repeated-bout effect and greater fascicle length?

The high and moderate responders did not differ in any of their baseline measurements, although there were differences in the levels of sport activity and training status (Table 1). In this regard, the moderate responders participated in athletics (participants 1 and 9) and football (participant 7), which involve sprint accelerations over different distances. Since hamstring eccentric force and activation are important in human sprint running mechanics (Kyröläinen et al., 2005; Schache et al., 2011, 2014; Morin et al., 2015; Sun et al., 2015), the practice of athletics and football might have produced a “repeated-bout effect” (RBE), which describes a protective adaptation occurring after a single bout of unaccustomed eccentric exercise that induces muscle damage (McHugh, 2003). Although adaptive mechanisms underlining the RBE remains unclear, recent well-supported theories suggest that eccentric exercises induce changes to connective tissue structures, such as extracellular matrix remodeling (Hoffman et al., 2016), and improves mitochondrial Ca^2+^ homeostasis, thereby stabilizing mitochondrial respiratory function (Rattray et al., 2013). In the present study, an enhancement of mitochondrial metabolism could have occurred since moderate responders showed no serum increases of sMtCK. Moreover, the chronic exposure to eccentric-biased exercise (i.e., sprint accelerations in athletics or football) might have induced hamstring muscles architectural changes in the moderate responders. Because sarcomere lengthening is non-uniform (Moo et al., 2016, 2017) and fascicles may have a regional response (i.e., more pronounced mechanical stretch might have been applied to single sarcomeres and thus fascicles) (Franchi et al., 2017), increases in fascicle length might be expected as an adaptive response to eccentric exercise. Those changes in fascicle length theoretically imply the addition of sarcomeres in series (Franchi et al., 2017), which may had a positive effect on eccentric hamstring strength at longer muscle lengths (Brockett et al., 2001) and produced a protective effect on the hamstring muscles (Timmins et al., 2016). While speculative, the RBE as well as hamstring muscles longer fascicle lengths as an adaptive mechanism to the type of sport practiced (eccentric exercises involving the hamstring muscles, e.g., sprinting), and the activity level (training status) could explain the smaller decline in hamstring muscle function and the low serum sMtCK levels in the moderate responders. The extraordinary tolerance to intensive eccentric exercise shown by moderate responders makes it necessary to analyze, in future studies, the extent of muscle damage in a group of eccentric resistance-trained subjects, from which a mild response might be expected (i.e., mild MVC decreases of <20% of the baseline values and recovery completed on 24–48 h) (Paulsen et al., 2012).

### Differences within subjects (limb-to-limb comparison)

Experimental designs based on an observational comparison of response using contralateral limbs in a single group have been applied to studies analyzing muscle damage (Mackey et al., 2008; Paulsen et al., 2010). This kind of experimental design is based on the assumption that changes in the markers of muscle damage between limbs are similar; however, our study also found significant differences in muscle function within subjects among the high responders. To the best of our knowledge, this is the first study comparing hamstring muscle damage within subjects (limb-to-limb comparison) in response to the same unilateral leg curl exercise performed with each leg. The high responders showed significant differences in the MVC reductions between legs after exercising, indicating that, contrary to our hypothesis, hamstring muscles (ST and, to a lesser extent, BFsh) from each leg suffered different degrees of damage. As observed in previous studies, these differences in the magnitude of EIMD were not influenced by lower limb dominance (Hody et al., 2013). One limitation in the present investigation was that no force or velocity data were recorded during the exercise. Therefore, although the same exercise load was placed on both legs and the subjects tried to maintain a lowering velocity that was as constant as possible during the 3-s eccentric phase, slightly different force levels could have been produced, particularly at the later stages of ELC due to fatigue. During the last set of the ELC regimen, the high responders could not maintain a constant lowering velocity over long muscle lengths. Since damage occurs when sarcomeres are actively stretched to lengths corresponding to that of the descending limb of the length-tension relationship (Morgan, 1990; Hoffman et al., 2016), the force and velocity production could be important, especially at long muscle lengths. Furthermore, no baseline differences were observed in the MVC between legs. However, baseline hamstring MVC was measured at an optimal sarcomere operating length (hip flexion, 40°; knee flexion, 30°) and baseline differences at long muscle lengths cannot be ruled out. Future investigations should carefully monitor force production during the range of motion or, at least, measure baseline MVC at longer muscle lengths.

Differences within subjects were not observed in the fMRI T2 results. The T2 data and MVC reductions correlated in all the ST regions measured in the leg with the greater FGC reduction of the hamstring muscles, but no such correlation was observed in the leg with the smaller FGC reduction. T2 values have been shown to remain elevated following eccentric exercises, even when other markers of muscle damage (i.e., FGC and serum enzyme levels) have returned to baseline values (Clarkson and Hubal, 2002). Indeed, T2 values have been found to remain elevated for as long as 75 days (Shellock et al., 1991) and 31 days (Nosaka and Clarkson, 1996) following (in both cases) eccentric exercising of the elbow flexor muscles. Foley et al. (1999) suggested that long-lasting T2 increases after other markers of EIMD have returned to baseline values might reflect an adaptive process rather than damage. In fact, T2 fMRI scans provide information on the metabolic state of the muscle fibers (Cagnie et al., 2011; Mendiguchia et al., 2013b), with the higher the T2 signal after an exercise, the higher the metabolic activity of the muscle (Schuermans et al., 2016). Since muscle function in the leg with the smaller FGC reduction of the hamstring muscles recovered 7 days after the exercise protocol (i.e., MVC returned to baseline values), it seems reasonable to link the long-lasting increases in T2 values to an enhanced remodeling of the ST and BFsh muscles, an adaptation consistent with protection against further damage. Although changes in T2 signals are a relevant marker of muscle damage (Larsen et al., 2007; Black and McCully, 2008; Fouré et al., 2015), our results indicate that fMRI must be accompanied by muscle function assessments to measure EIMD.

## Summary and conclusion

The present study explored individual and regional ST, BFlh and BFsh damage in response to an intensive ELC regimen performed with each leg, analyzing differences in the extent of EIMD between and within subjects (limb-to-limb comparison). The proxy markers used enabled the characterization of the EIMD suffered by the ST and BF muscles following ELCs. Changes in the FGC and serum enzyme levels measured in 10 of the subjects (*n* = 13) indicated severe muscle damage. However, the results showed a wide range of peak MVC reductions among the subjects (from 7 to 84%), reflecting extremely different degrees of damage between the subjects (high and moderate responders) that may be due to the protective RBE as well as hamstring muscles longer fascicles length as an adaptive mechanism to the type of sport practiced (eccentric exercises involving the hamstring muscles, e.g., sprinting), and the activity level (training status) of some of the participants. fMRI analysis confirmed that the ST was the hamstring muscle most damaged by ELCs in the high responders. Despite the potential protective role of the tendinous inscription described by previous studies, uniform T2 changes were observed across all the measured sections of this muscle. Since the ST muscle could suffer an anomalous recruitment pattern due to fatigue and damage, an excessive load would be placed on the BFsh, causing it to perform a synergistic compensation that leads to structural damage. Finally, T2 and MVC values did not correlate for the leg with the smaller FGC reduction of the hamstring muscles. These results suggest that long-lasting T2 increases after FGC markers have returned to baseline values (functional recovery of the hamstring muscles) might reflect an adaptive process rather than damage.

## Author contributions

GC, JM, XA, JP, LN, GR, RB, and JC: designed the experiments; GC, XA, JP, DS, LN, GR, RB, and JC: performed the experiments; GC, JM, XA, and JC: analyzed the data; GC, JM, XA, and JC: interpreted the results; GC, JM, and JC: drafted the manuscript and prepared the tables and figures; GC, JM, XA, JP, DS, LN, GR, RC, RB, and JC: revised the paper and approved the final version of the manuscript.

### Conflict of interest statement

GR was employed by company FC Barcelona. JM was employed by company Zentrum Rehab and Performance Center. All other authors declare no competing interests.
